# Supplementary material for: Policy and programmatic directions for the Lesotho tuberculosis programme: Findings of the national tuberculosis prevalence survey, 2019
Source: PLoS One. 2023 Mar 9;18(3):e0273245. doi: 10.1371/journal.pone.0273245 (PMC9997977; doi:10.1371/journal.pone.0273245)
Supplement: Attachment — Submitted filename: Response to reviewers July 28.docx [file pone.0273245.s001.docx]

**Response to reviewers**

28 July 2022

Dear Editor,

We would like to thank you for your feedback on our earlier draft. We are hereby submitting the final manuscript for your review.

1. We have deleted all old files and in this submission are only including the final version of the manuscript that incorporates earlier requested revisions.
2. The map included in this document was developed by the Survey Team with the Lesotho Office of Statistics while selecting the sampling units.

Please let us know if you have any further questions.

Kind regards,

Neeraj Kak
